# Prognostic value of extrahepatic metastasis on colon cancer with liver metastasis: a retrospective cohort study

**DOI:** 10.3389/fonc.2023.1172670

**Published:** 2023-06-06

**Authors:** Shuheng Bai, Ling Chen, Guixian Zhu, Wang Xuan, Fengyuan Hu, Wanyi Liu, Wenyang Li, Ning Lan, Min Chen, Yanli Yan, Rong Li, Yiping Yang, Juan Ren

**Affiliations:** ^1^ Department of Radiotherapy, The First Affiliated Hospital of Xi’an Jiaotong University, Xi’an, China; ^2^ Department of Chemotherapy, The First Affiliated Hospital of Xi’an Jiaotong University, Xi’an, China; ^3^ Department of Oncology, The Second Affiliated Hospital of Xi’an Jiaotong University, Xi’an, China; ^4^ Department of Radiotherapy, Radiotherapy Clinical Medical Research Center of Shaanxi Province, Xi’an, China

**Keywords:** colorectal cancer, competing risk analysis, extrahepatic metastasis, prognostic model, SEER, risk factor

## Abstract

**Introduction:**

The occurrence of metastasis is a threat to patients with colon cancer (CC), and the liver is the most common metastasis organ. However, the role of the extrahepatic organs in patients with liver metastasis (LM) has not been distinctly demonstrated. Therefore, this research aimed to explore the prognostic value of extrahepatic metastases (EHMs).

**Methods:**

In this retrospective study, a total of 13,662 colon patients with LM between 2010 and 2015 were selected from the Surveillance, Epidemiology, and End Results database (SEER). Fine and Gray’s analysis and K–M survival analysis were utilized to explore the impacts of the number of sites of EHMs and different sites of EHMs on prognosis. Finally, a prognostic nomogram model based on the number of sites of EHMs was constructed, and a string of validation methods was conducted, including concordance index (C-index), receiver operating characteristic curves (ROC), and decision curve analysis (DCA).

**Results:**

Patients without EHMs had better prognoses in cancer-specific survival (CSS) and overall survival (OS) than patients with EHMs (*p* < 0.001). Varied EHM sites of patients had different characteristics of primary location site, grade, and histology. Cumulative incidence rates for CSS surpassed that for other causes in patients with 0, 1, 2, ≥ 3 EHMs, and the patients with more numbers of sites of EHMs revealed worse prognosis in CSS (*p* < 0.001). However, patients with different EHM sites had a minor difference in cumulative incidence rates for CSS (*p* = 0.106). Finally, a nomogram was constructed to predict the survival probability of patients with EHMs, which is based on the number of sites of EHMs and has been proven an excellent predictive ability.

**Conclusion:**

The number of sites of EHMs was a significant prognostic factor of CC patients with LM. However, the sites of EHMs showed limited impact on survival. Furthermore, a nomogram based on the number of sites of EHMs was constructed to predict the OS of patients with EHMs accurately.

## Introduction

Colorectal cancer (CRC) leads to a vast health program in the world, with an incidence of 10.2% and a mortality of 9.2% all over the world in 2018, which is the third most common cancer and the second leading cause of cancer-related mortality (1). Moreover, the incidence and deaths are increasing for both males and females in most countries, especially in developing countries (2). CRC can be divided into colon cancer (CC), and rectum cancer. CC, the most common of these subtypes, was responsible for 59.46% of new cases and 61.68% of fatalities globally in 2020. Additionally, among all malignancies, CC alone ranks seventh in terms of new cases and fatalities (2, 3).

The occurrence of metastasis is a threat for CRC patients and may be fatal (4). At the time of first diagnosis, 20% of CRC patients had metastases, and this percentage has remained constant over the past two decades (5). The prognosis for CRC patients with distant metastases is substantially worse, and the 5-year survival rates for CRC patients with or without localized tumors are 91 and 14%, respectively (6). The liver is the most common organ for distant metastasis of CRC with a relatively poor prognosis (7). Population-based research reported that 24.7% of patients with CRC developed liver metastasis (LM) during the disease. About 71% of patients with LM were found at first diagnosis (8). The 5-year survival rate is lower than 5% for these patients with only palliative intent (9). However, in patients who have received successful radical resection of LM, a 5-year survival rate can achieve to 30–57% (10–12).

Great effort has been made to predict how CRC patients with LM would do. AJCC stage system is a reliable criterion for evaluating patients’ prognoses. However, it is unsuitable for patients with distant metastasis (Stage IV), especially patients with more than one metastasis organ. Some population-based research constructed a prognostic model for CRC with distant metastasis (13, 14). Nevertheless, the number of sites of extrahepatic metastases (EHMs) was not involved in these prognostic analyses, which may be an efficient indicator for the survival of patients with LM. To our best research, the role of the number of extrahepatic organs in LM has not been clearly demonstrated. Additionally, a certain extrahepatic organ metastasis may result in a particular prognosis. It may be efficient to improve the prognostic value of these models when considering the information on the number of sites of EHMs and metastatic sites.

Thus, in this research, we mainly investigated the prognostic values of the number of sites of EHMs and explored the prognostic features among different EHM sites. Moreover, in order to estimate survival time, we also created a unique risk framework, and the nomogram was validated in a validation cohort.

## Methods

### Data acquisition and eligibility criteria

This was a retrospective research employing information from the Surveillance, Epidemiology, and End Results (SEER) database (https://seer.cancer.gov/data/), which is a public cancer statistic database founded by the National Cancer Institute (NCI), providing a string of clinical and pathological data including treatment, metastasis, and survival time of many tumors. In this present study, data from patients were extracted by utilizing SEER*Stat (version 8.4.0). The requirement of the Declaration of Helsinki was honored in this research.

In this study, individuals with LM of the colon who were diagnosed between 2010 and 2015 were included after meeting certain inclusion and exclusion criteria. The inclusion criteria were as follows: (a) malignancies that originated in colon without rectum, (b) patients with confirmed pathological diagnosis (biopsy or surgical samples), and (c) patients with confirmed LM from CC. Exclusion criteria were as follows: (a) patients with unknown metastasis information, (b) evidence of other coexisting malignancies, and (c) patients without complete records for American Joint Committee on Cancer (AJCC)-TNM staging, treatment, overall survival (OS), and cancer-specific survival (CSS).

### Variable extraction and cohort identification

Clinical variables were extracted from this dataset, including demographic features, location site of the primary lesion, grade, T stage, N stage, M stage, detailed information about metastasis, histology, anticancer treatment (surgery, radiotherapy, and chemotherapy), survival status, and survival time (CSS and OS). The location of the primary lesion was further divided into the right-sided colon and left-sided colon. The histology was classified into four subtypes: adenocarcinoma, mucinous adenocarcinoma, signet ring cell carcinoma, and other. Furthermore, T stage fell into two categories: “T1-2” and “T3-4.” In this research, all eligible patients were randomly divided into a training cohort and a validation cohort according to a ratio of 7:3 before constructing our nomogram.

### Statistical analysis

All categorical variables were summarized as count and percentage, and Chi-square test and Fisher’s exact test were utilized for categorized data. The clinical and demographic features of eligible patients with involvement of 0, 1, 2, or 3 extrahepatic organs were compiled using descriptive statistics. Kaplan–Meier analysis was performed by utilizing the “survival” and “survmine” packages in R software, and a Log-Rank test was used to assess how the curves differed from one another. By using the R software’s “Survival” package, univariate and multivariate regression analyses were used to pinpoint OS and CSS prognostic risk variables. Using the “cmprsk” package in R, the Fine and Gray’s model was adjusted to forecast the cumulative incidence function (CIF) of mortality from CSS and other causes. The “rms” R program was used to create the nomogram for our model. Moreover, a string of validation measurements, including concordance index (Cindex), receiver operating characteristic curves (ROC), and calibration curves, were conducted to evaluate the discrimination ability and calibration ability of our nomogram by utilizing “riskRegression,” “timeROC,” “pec,” “cmprsk,” and “survival” R packages. In this study, R software (version 4.1.2) was adopted for all statistical analyses. *P* value < 0.05 was considered statistically significant, and all statistical tests were two-sided.

## Results

### Clinical characteristics of eligible patients

A total of 13,662 CC patients with LM were enrolled in our research, among whom 6,262 were women and 7,400 were men. For the distribution of the number of EHM in our overall cohort, 10,131 (74%) patients had zero involved extrahepatic organs; 2,766 (20%) patients had one involved extrahepatic organ; 673 (4.9%) patients had two involved extrahepatic organs, and 92 (0.7%) patients had three or more than three involved extrahepatic organs. Detailed information about other clinical features of the overall cohort was summarized in **Table 1**.

**Table 1:** Clinicopathological characteristics between training cohort and validation cohort.

Variable	*N*	Overall, *N* = 13,662	Train cohort, *N* = 9,628	Test cohort, *N* = 4,034	*p*-value
Sex	13,662				> 0.9
Female		6,262 (46%)	4,412 (46%)	1,850 (46%)	
Male		7,400 (54%)	5,216 (54%)	2,184 (54%)	
Age	13,662				0.2
1–49		1,893 (14%)	1,338 (14%)	555 (14%)	
50–59		2,973 (22%)	2,104 (22%)	869 (22%)	
60–69		3,651 (27%)	2,583 (27%)	1,068 (26%)	
70–79		2,868 (21%)	1,971 (20%)	897 (22%)	
80+		2,277 (17%)	1,632 (17%)	645 (16%)	
Race	13,662				0.3
White		10,236 (75%)	7,204 (75%)	3,032 (75%)	
Black		2,318 (17%)	1,658 (17%)	660 (16%)	
Other		1,108 (8.1%)	766 (8.0%)	342 (8.5%)	
Marital.status	13,662				0.4
Married		7,099 (52%)	5,030 (52%)	2,069 (51%)	
Unmarried		5,948 (44%)	4,158 (43%)	1,790 (44%)	
Unknown		615 (4.5%)	440 (4.6%)	175 (4.3%)	
primary.location	13,662				0.7
Left		6,050 (44%)	4,253 (44%)	1,797 (45%)	
Right		7,612 (56%)	5,375 (56%)	2,237 (55%)	
Histology	13,662				> 0.9
Adenocarcinoma		11,808 (86%)	8,326 (86%)	3,482 (86%)	
Mucinous adenocarcinoma		867 (6.3%)	613 (6.4%)	254 (6.3%)	
Signet ring cell carcinoma		80 (0.6%)	54 (0.6%)	26 (0.6%)	
Other		907 (6.6%)	635 (6.6%)	272 (6.7%)	
Grade	13,662				0.6
Grade I		552 (4.0%)	391 (4.1%)	161 (4.0%)	
Grade II		7,793 (57%)	5,516 (57%)	2,277 (56%)	
Grade III		2,827 (21%)	1,957 (20%)	870 (22%)	
Grade IV		662 (4.8%)	463 (4.8%)	199 (4.9%)	
Unknown		1,828 (13%)	1,301 (14%)	527 (13%)	
pT	13,662				0.9
T1		1,822 (13%)	1,287 (13%)	535 (13%)	
T2		378 (2.8%)	260 (2.7%)	118 (2.9%)	
T3		6,437 (47%)	4,550 (47%)	1,887 (47%)	
T4		5,025 (37%)	3,531 (37%)	1,494 (37%)	
pN	13,662				0.4
N0		3,721 (27%)	2,602 (27%)	1,119 (28%)	
N1		5,133 (38%)	3,652 (38%)	1,481 (37%)	
N2		4,808 (35%)	3,374 (35%)	1,434 (36%)	
Radiotherapy	13,662				0.8
None		13,171 (96%)	9,279 (96%)	3,892 (96%)	
Performed		491 (3.6%)	349 (3.6%)	142 (3.5%)	
Cheomtherapy	13,662				0.12
None		4,698 (34%)	3,271 (34%)	1,427 (35%)	
Performed		8,964 (66%)	6,357 (66%)	2,607 (65%)	
Surgery	13,662				>0.9
None		3,168 (23%)	2,231 (23%)	937 (23%)	
Performed		10,494 (77%)	7,397 (77%)	3,097 (77%)	
CEA	13,662				0.3
Negative		1,485 (11%)	1,061 (11%)	424 (11%)	
Positive		7,972 (58%)	5,637 (59%)	2,335 (58%)	
Unknown		4,205 (31%)	2,930 (30%)	1,275 (32%)	
extrehepatic.metastates.number	13,662				0.7
0		10,131 (74%)	7,136 (74%)	2,995 (74%)	
1		2,766 (20%)	1,945 (20%)	821 (20%)	
2		673 (4.9%)	485 (5.0%)	188 (4.7%)	
≥3		92 (0.7%)	62 (0.6%)	30 (0.7%)	

^1^n (%).

^2^ Pearson’s Chi-squared test.

In our research, we also divided all patients into train-cohort and test-cohort randomly according to a ratio of 7:3 for our prognostic model’s construction and validation subsequently. Moreover, we found no significant statistical difference among clinicopathological characteristics between these two cohorts *via* the Chi-square test (**Table 1**).

### The correlation between clinicopathological characteristics and the number of extrahepatic metastasis sites

As shown in **Table 2**, CC patients with different numbers of EHMs presented a significant difference in some clinicopathological features, including age, grade, T stage, N stage, CEA, radiotherapy, and surgery (all *p* < 0.05). However, other characteristics did not present an obvious association with the number of EHM. It is worth noting that patients with a larger number of EHMs were more likely to have a higher rate of N1 stage (the percentages of patients with N1 stage in patients with none EHM site *vs.* patients with 1 EHM site *vs.* patients with 2 EHM sites *vs.* patients with ≥3 EHM sites: 37% *vs.* 39% *vs.* 44% *vs.* 45%, *p* < 0.001), nevertheless, a lower rate of N0 stage (the percentages of patients with N0 stage in patients with none EHM site *vs.* patients with 1 EHM site *vs.* patients with 2 EHM sites *vs.* patients with ≥ 3 EHM sites: 27% *vs.* 27% *vs.* 26% *vs.* 21%, *p* < 0.001). Meanwhile, we also found an interesting phenomenon that patients with a more significant number of EHM sites were more likely to present a positive result of CEA (the percentages of patients with positive CEA in patients with none EHM site *vs.* patients with 1 EHM site *vs.* patients with 2 EHM sites *vs.* patients with ≥ 3 EHM sites: 56% *vs.* 63% *vs.* 67% *vs.* 72%, *p* < 0.001); however, the opposite result was presented in CEA negative (12% *vs.* 8.5% *vs.* 6.8% *vs.* 2.2%, *p* < 0.001). For the therapy aspect, patients with a smaller number of EHMs were more likely to accept surgery and radiotherapy. Nevertheless, there were no differences in the amount of ECM across chemotherapy patients.

**Table 2:** Characteristics of CC patients with liver metastasis.

		The number of involved extrahepatic organs				
Variable	Overall, *N* = 13,662	0, *N* = 10,131	1, *N* = 2,766	2, *N* = 673	>= 3, *N* = 92	*p*-value
Sex						0.1
Female	6,262 (46%)	4,670 (46%)	1,272 (46%)	277 (41%)	43 (47%)	
Male	7,400 (54%)	5,461 (54%)	1,494 (54%)	396 (59%)	49 (53%)	
Age						0.001
1–49	1,893 (14%)	1,386 (14%)	398 (14%)	96 (14%)	13 (14%)	
50–59	2,973 (22%)	2,161 (21%)	624 (23%)	158 (23%)	30 (33%)	
60–69	3,651 (27%)	2,670 (26%)	762 (28%)	193 (29%)	26 (28%)	
70–79	2,868 (21%)	2,138 (21%)	575 (21%)	139 (21%)	16 (17%)	
80+	2,277 (17%)	1,776 (18%)	407 (15%)	87 (13%)	7 (7.6%)	
Race						0.054
White	10,236 (75%)	7,652 (76%)	2,021 (73%)	491 (73%)	72 (78%)	
Black	2,318 (17%)	1,687 (17%)	503 (18%)	113 (17%)	15 (16%)	
Other	1,108 (8.1%)	792 (7.8%)	242 (8.7%)	69 (10%)	5 (5.4%)	
Marital status						0.19
Married	7,099 (52%)	5,275 (52%)	1,423 (51%)	343 (51%)	58 (63%)	
Unmarried	5,948 (44%)	4,391 (43%)	1,232 (45%)	293 (44%)	32 (35%)	
Unknown	615 (4.5%)	465 (4.6%)	111 (4.0%)	37 (5.5%)	2 (2.2%)	
Primary location						0.7
Left	6,050 (44%)	4,461 (44%)	1,242 (45%)	308 (46%)	39 (42%)	
Right	7,612 (56%)	5,670 (56%)	1,524 (55%)	365 (54%)	53 (58%)	
Histology						0.18
Adenocarcinoma	11,808 (86%)	8,767 (87%)	2,376 (86%)	588 (87%)	77 (84%)	
Mucinous adenocarcinoma	867 (6.3%)	658 (6.5%)	170 (6.1%)	31 (4.6%)	8 (8.7%)	
Signet ring cell carcinoma	80 (0.6%)	55 (0.5%)	17 (0.6%)	7 (1.0%)	1 (1.1%)	
Other	907 (6.6%)	651 (6.4%)	203 (7.3%)	47 (7.0%)	6 (6.5%)	
Grade						< 0.001
Grade I	552 (4.0%)	428 (4.2%)	102 (3.7%)	18 (2.7%)	4 (4.3%)	
Grade II	7,793 (57%)	5,983 (59%)	1,449 (52%)	329 (49%)	32 (35%)	
Grade III	2,827 (21%)	2,093 (21%)	565 (20%)	148 (22%)	21 (23%)	
Grade IV	662 (4.8%)	500 (4.9%)	137 (5.0%)	20 (3.0%)	5 (5.4%)	
Unknown	1,828 (13%)	1,127 (11%)	513 (19%)	158 (23%)	30 (33%)	
pT						< 0.001
T1-2	2,200 (16%)	1,431 (14%)	562 (20%)	185 (27%)	22 (24%)	
T3-4	11,462 (84%)	8,700 (86%)	2,204 (80%)	488 (73%)	70 (76%)	
pN						0.003
N0	3,721 (27%)	2,777 (27%)	749 (27%)	176 (26%)	19 (21%)	
N1	5,133 (38%)	3,725 (37%)	1,073 (39%)	294 (44%)	41 (45%)	
N2	4,808 (35%)	3,629 (36%)	944 (34%)	203 (30%)	32 (35%)	
Radiotherapy						< 0.001
None	13,171 (96%)	9,880 (98%)	2,633 (95%)	599 (89%)	59 (64%)	
Performed	491 (3.6%)	251 (2.5%)	133 (4.8%)	74 (11%)	33 (36%)	
Chemotherapy						0.3
None	4,698 (34%)	3,521 (35%)	913 (33%)	235 (35%)	29 (32%)	
Performed	8,964 (66%)	6,610 (65%)	1,853 (67%)	438 (65%)	63 (68%)	
Surgery						< 0.001
None	3,168 (23%)	1,787 (18%)	977 (35%)	349 (52%)	55 (60%)	
Performed	10,494 (77%)	8,344 (82%)	1,789 (65%)	324 (48%)	37 (40%)	
CEA						< 0.001
Negative	1,485 (11%)	1,201 (12%)	236 (8.5%)	46 (6.8%)	2 (2.2%)	
Positive	7,972 (58%)	5,703 (56%)	1,749 (63%)	454 (67%)	66 (72%)	
Unknown	4,205 (31%)	3,227 (32%)	781 (28%)	173 (26%)	24 (26%)	

^1^n (%).

^2^Pearson’s Chi-squared test; Fisher’s exact test.

### Extrahepatic metastasis sites

**Figure 1:**
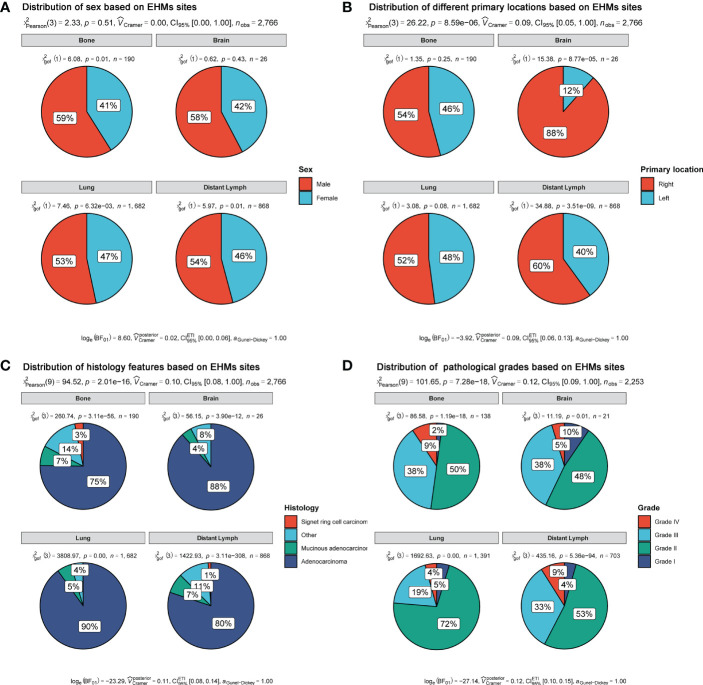
Distribution of clinical features based on EHM sites. **(A)** Sex feature. **(B)** Primary location. **(C)** Histology feature. **(D)** Pathological grades.

In our cohort, the most and least frequent EHM sites were the lung (57.54%) and brain (2.31%), respectively. Moreover, the frequent EHM sites of distant lymph nodes (dLNs) and bone were 11.6 and 35.46%, respectively. Then, we selected patients with only one EHM site to explore whether different EHM sites presented various patterns of some clinical features, including sex, primary location site, grade, and histology. As **Figure 1** shown, patients with various EHM sites presented different distributed characteristics significantly for primary location site, grade, and histology. We easily found that the primary location on the right accounted for the highest rate (88%) of brain metastasis, and the rate was higher than other metastasis sites. Patients with lung metastasis had the highest rate of adenocarcinoma (90%). Nevertheless, patients with bone metastasis had the lowest (75%).

Moreover, it is notable that patients of Grade II accounted for the most prominent rate in all different metastases sites, rather than patients of Grade IV or III. Meanwhile, patients with lung metastasis had different frequencies for grade compared with patients with others metastasis. Grade III+IV accounted for only 23% of patients with lung metastasis. However, the rates of grade III+IV were 47, 43, and 42% in patients with metastases of bone, brain, and dLNs, respectively.

### The prognostics value of the number of sites of extrahepatic metastases

**Figure 2:**
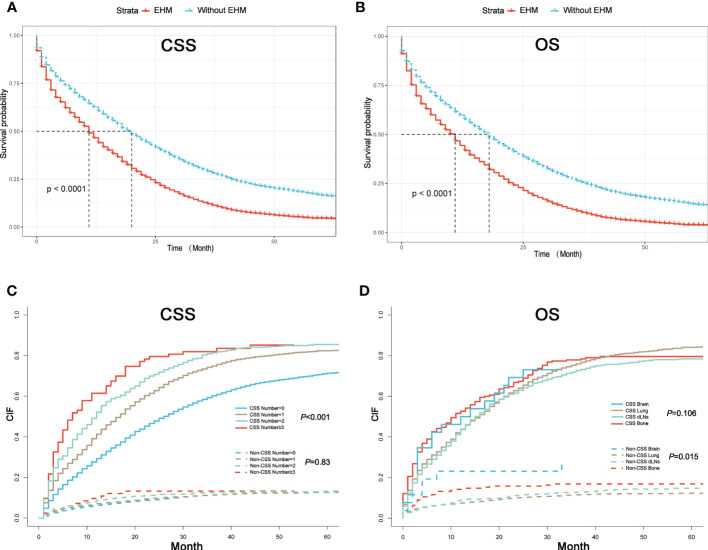
**(A)** K–M curves of CSS for patients with or without extrahepatic metastases (EHMs). **(B)** K–M curves of OS for patients with or without EHMs. **(C)** Cumulative incidence function curves of CSS and non-CSS cause according to the number of sites of EHMs. **(D)** Cumulative incidence function curves of CSS and non-CSS cause according to different sites of EHMs.

We first explored whether the presence of EHMs had an impact on prognosis. As shown in **Figures 2A, B**, patients without EHMs had better prognosis both in CSS and OS than patients with others who had EHMs [median CSS: 20 months (without EHMs) *vs.* 11 months (with EHMs), *p* < 0.001; median OS: 18 months (without EHMs) *vs.* 11 months (with EHMs), *p* < 0.001].

**Figure 3:**
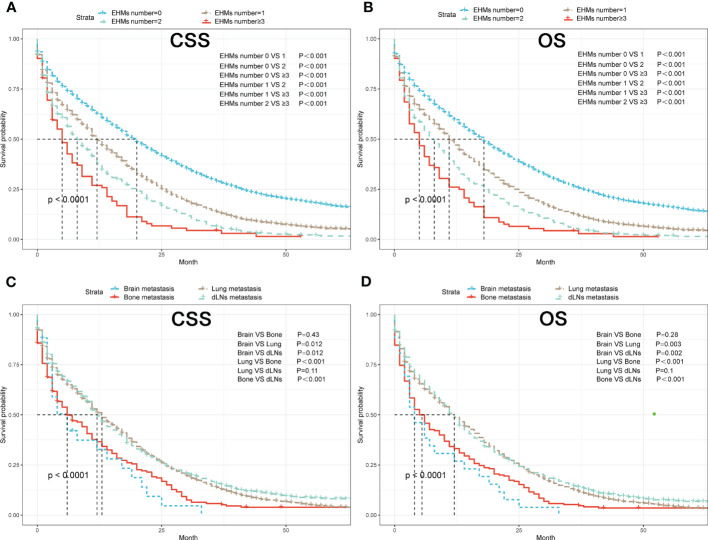
**(A)** K–M curves of CSS for patients with different numbers of extrahepatic metastases (EHMs). **(B)** K–M curves of OS for patients with different numbers of EHMs. **(C)** K–M curves of CSS for patients with different sites of EHMs. **(D)** K–M curves of OS for patients with different sites of EHMs.

**Figure 4:**
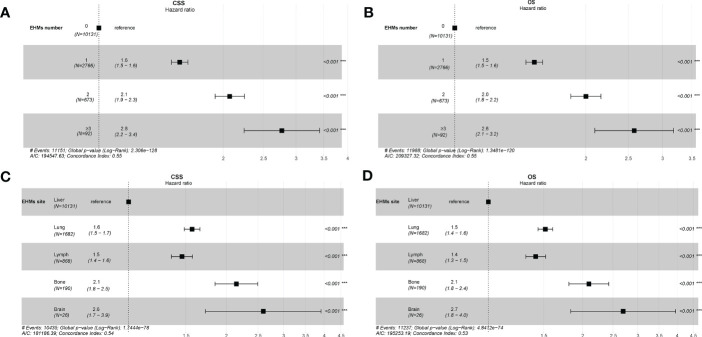
**(A)** Univariate cox regression analysis of CSS for different numbers of extrahepatic metastases (EHMs). **(B)** Univariate cox regression analysis of overall survival (OS) for different numbers of EHMs. **(C)** Univariate cox regression analysis of CSS for different sites of EHMs. **(D)** Univariate cox regression analysis of OS for different sites of EHMs.

The cumulative incidence rates for CSS outnumbered those for other causes in patients with 0, 1, 2, or ≥ 3 EHMs, and cumulative incidence rates for CSS were increased gradually with time since initial (**Figure 2C**). In addition, the patients with a larger number of EHMs revealed worse prognosis in CSS (*p* < 0.001); however, it is not different in other causes (*p* = 0.83). Moreover, we found that the CSS and OS were correlated with the number of sites of EHMs significantly [median CSS: 20 months *vs.* 12 months *vs.* 8 months *vs.* 5 months (EHMs number: 0 *vs.* 1 *vs.* 2 *vs.* ≥3), *p* < 0.001; median CSS: 18 months *vs.* 11 months *vs.* 8 months *vs.* 5 months (EHMs number: 0 *vs.* 1 *vs.* 2 *vs.* ≥3)] by utilizing K-M analysis for survival rates (**Figures 3A, B**). Univariate cox regression analysis was also conducted to explore the prognostic value of the number EHMs. As shown in **Figures 4A, B**, patients with a larger number of EHMs presented a higher hazard ratio (HR) for CSS and OS [CSS-HR: 1.6(1 *vs.* 0), 2.1(2 *vs.* 0), 2.8 (≥3 *vs.* 0); OS-HR: 1.5(1 *vs.* 0), 2.0(2 *vs.* 0), 2.6 (≥ 3 *vs.* 0), all *p* < 0.001]. In addition, multivariate cox regression analysis showed a consistent result after adjustment for age, race, gender, histology, marital status, grade, CEA, and stage N and T, and treatment [CSS-HR: 1.44(1 *vs.* 0), 1.61(2 *vs.* 0), 2.12 (≥ 3 *vs.* 0); OS-HR: 1.41(1 *vs.* 0), 1.57(2 *vs.* 0), 2.03 (≥3 *vs.* 0), all *p* < 0.001] (**Table 3**).

**Table 3:** Multivariate cox regression analysis on clinical variables including EHMs.

	CSS			OS		
Characteristic	HR^1^	95% CI^1^	*p*-value	HR^1^	95% CI^1^	*p*-value
EHMs number						
0	—	—		—	—	
1	1.44	1.37, 1.51	< 0.001	1.41	1.34, 1.47	< 0.001
2	1.61	1.48, 1.75	< 0.001	1.57	1.45, 1.71	< 0.001
≥ 3	2.12	1.71, 2.63	< 0.001	2.03	1.64, 2.51	< 0.001
Sex						
Female	—	—		—	—	
Male	1.09	1.04, 1.13	< 0.001	1.1	1.06, 1.14	< 0.001
Age						
1–49	—	—		—	—	
50–59	1.04	0.98, 1.11	0.2	1.06	0.99, 1.13	0.093
60–69	1.1	1.03, 1.17	0.003	1.13	1.07, 1.20	< 0.001
70–79	1.36	1.28, 1.46	< 0.001	1.43	1.34, 1.53	< 0.001
80+	1.78	1.66, 1.91	< 0.001	1.88	1.75, 2.01	< 0.001
Race						
Black	—	—		—	—	
Other	0.83	0.77, 0.90	< 0.001	0.83	0.76, 0.89	< 0.001
White	0.88	0.84, 0.93	< 0.001	0.87	0.83, 0.91	< 0.001
Marital status						
Married	—	—		—	—	
Unknown	0.99	0.90, 1.08	0.8	1.02	0.93, 1.11	0.7
Unmarried	1.11	1.07, 1.16	< 0.001	1.12	1.08, 1.16	< 0.001
Primary location						
Left	—	—		—	—	
Right	1.17	1.13, 1.22	< 0.001	1.16	1.12, 1.21	< 0.001
Histology						
Adenocarcinoma	—	—		—	—	
Mucinous adenocarcinoma	1.14	1.06, 1.23	< 0.001	1.13	1.05, 1.22	0.001
Other	1.19	1.09, 1.29	< 0.001	1.23	1.14, 1.33	< 0.001
Signet ring cell carcinoma	1.42	1.12, 1.80	0.004	1.46	1.16, 1.84	0.001
Grade						
Grade I	—	—		—	—	
Grade II	1.38	1.24, 1.53	< 0.001	1.39	1.26, 1.53	< 0.001
Grade III	2.09	1.88, 2.33	< 0.001	2.07	1.87, 2.30	< 0.001
Grade IV	2.4	2.11, 2.73	< 0.001	2.37	2.09, 2.68	< 0.001
Unknown	1.7	1.52, 1.91	< 0.001	1.73	1.55, 1.93	< 0.001
pT						
T1-2	—	—		—	—	
T3-4	1.07	1.01, 1.14	0.027	1.06	1.00, 1.13	0.036
pN						
N0	—	—		—	—	
N1	1.15	1.10, 1.21	< 0.001	1.15	1.09, 1.20	< 0.001
N2	1.64	1.55, 1.74	< 0.001	1.59	1.51, 1.68	< 0.001
Radiotherapy						
None	—	—		—	—	
Performed	1.03	0.93, 1.14	0.6	1.02	0.92, 1.12	0.7
Cheomtherapy						
None	—	—		—	—	
Performed	0.37	0.36, 0.39	< 0.001	0.36	0.35, 0.38	< 0.001
Surgery						
None	—	—		—	—	
Performed	0.39	0.36, 0.41	< 0.001	0.4	0.38, 0.42	< 0.001
CEA						
Negative	—	—		—	—	
Positive	1.52	1.42, 1.62	< 0.001	1.52	1.42, 1.62	< 0.001
Unknown	1.4	1.31, 1.50	< 0.001	1.42	1.33, 1.52	< 0.001

^1^HR, hazard ratio; CI, confidence interval; —, Reference.

### The prognostic difference among variant extrahepatic metastasis sites

To further understand whether different EHM sites impacted survival time, we also compared CSS and OS in patients with bong, lung, dLNs, and brain as the only EHMs organ. As **Figure 2B** shown, patients with different EHM sites had a more negligible difference in cumulative incidence rates for CSS (*p* = 0.106). However, patients with brain metastasis presented the highest cumulative incidence rate for other causes (*p* = 0.015). In addition, as shown in **Figures 3C, D**, we found that brain metastasis and bone metastasis brought worse prognosis than lung metastasis and dLNs [CSS: 6 months *vs.* 13 months *vs.* 12 months (bone *vs.* brain *vs.* lung *vs.* dLNs), *p* > 0.05(bone *vs.* brain; lung *vs.* dLNs) other *p* < 0.05; OS: 5.5 months *vs.* 4 *vs.* 12 months *vs.* 12 months (bone *vs.* brain *vs.* lung *vs.* dLNs), *p* > 0.05(Bone *vs.* brain; lung *vs.* dLNs) other *p* < 0.05]. Moreover, Univariate cox regression analysis showed that EHMs presented a higher hazard ratio (HR) for CSS and OS (**Figures 4C, D**), and HRs of bone and brain were more severe that lung and dLNs (*p* < 0.001), which was consisted with our other results above.

### Construction and validation of nomogram for patients with extrahepatic metastases

**Figure 5:**
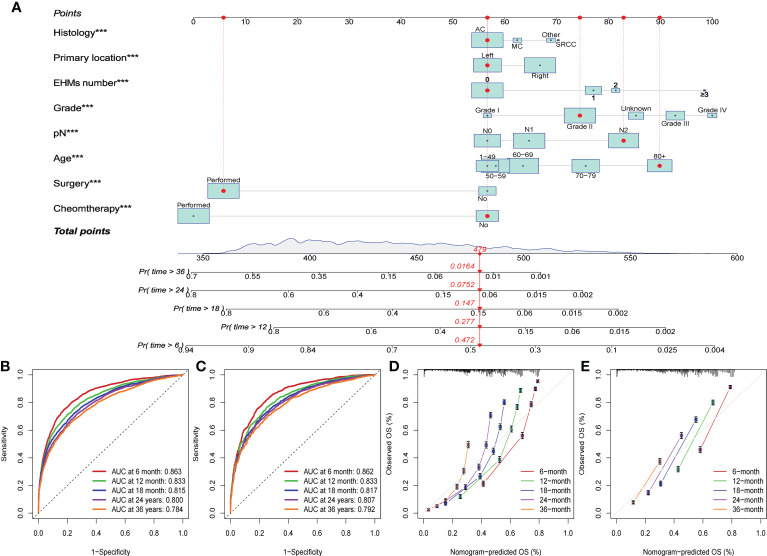
**(A)** The constructed nomogram for predicting 6-, 12-, 18-, 24-, and 36-month overall survival (OS) of patients with EHMs. ***: p≤0.001. **(B)** The ROC curves for predicting 6-, 12-, 18-, 24-, and 36-month OS in the training cohort. **(C)** The ROC curves for predicting 6-, 12-, 18-, 24-, and 36-month OS in the validation cohort. **(D)** The calibration curves for predicting 6-, 12-, 18-, 24-, and 36-month OS in the training cohort. **(E)** The calibration curves for predicting 6-, 12-, 18-, 24-, and 36-month OS in the validation cohort.

Finally, we constructed a nomogram to predict the survival probability of patients with EHMs (**Figure 5A**). EHMs number, age, primary location, histology, grade, CEA, stage N, and treatments were involved in this nomogram, which were employed to predict the total point of each patient, thus predicting the 6-, 12-, 18-, 24-, and 36-month OS probability of these patients. In addition, we calculated the C-index of this nomogram to estimate its predictive power, suggesting the model had excellent performance in predicting the OS of CC patients with EHMs (training cohort: 0.738; validation cohort: 0.739). Moreover, ROC curves and calibration curves were generated of our nomogram. As shown in **Figure 5B**, the area under the curve (AUC) of our nomogram were 0.863, 0.833, 0.815, 0.8, and 0.784 in predicting 6-, 12-, 18-, 24-, and 36- month OS, respectively, of patients in training cohort. Moreover, the AUCs of validation cohort were 0.862, 0.833, 0.817, 0.807, and 0.792, respectively (**Figure 5C**). Additionally, we displayed the calibration curves, which showed excellent concordance between the actual and projected OS in the training cohort and validation cohort (**Figures 5D, E**). All of these findings showed how well our nomogram predicted the likelihood that patients with EHMs would survive.

## Discussion

The liver is the most common distant metastatic organ in patients with CC and threaten patients’ survival time (15–17). Our results also revelated that CC patients with LM presented high cancer mortality. Some clinicopathological features affect patients’ prognosis, including TNM stage, age, TP53, KRAS, MSI, and so on (18–21). However, the way of EHMs impacts the prognosis of CC is still unclearly.

We found that the number of sites of EHMs was a significant prognostic factor for CC patients with LM. In our research, the patient with no EHMs had a better prognosis, while a patient with 1, 2, ≥ 3 EHMs presented a worse survival. It might result from that patient with EHMs would not receive liver resection. A study of 1,600 patients demonstrated that 5-year survival rates of patients who received liver resection could achieve above 50%, compared with only 5% for patients treated with palliative (10). Moreover, the survival time decreased as the number of EHMs increased, which may be attributed to the limited effectiveness of systemic treatment for patients with multiple metastases. A similar result was obtained by other research. Wang et al. found that more numbers of sites of EHMs were associated with poor prognosis in NSCLC patients with brain metastasis (22). Meanwhile, the results of cox regression analysis also implied that the number of sites of EHMs caused different hazard ratios significantly, which implied that it might be a suitable prognostic indicator for patients with EHMs.

Moreover, we also explored the impact of sites of EHMs on survival. In our research, patients with different EHM sites had a smaller difference in cumulative incidence rates for CSS, which indicted that the site of EHMs could not be a suitable index for prediction of survival time. Also, patient with brain metastasis and bone metastasis tended to present a worse prognosis than with lung metastasis and dLNs, which was consistent with other research. Tokore had reported that the median survival time of CC patient with brain metastasis was only 2.8 months (23). Moreover, the median survival time of bone metastasis was similar to brain metastasis, which was 5–7 months (24). Compared with the worse prognosis of brain and bone metastasis, the median survival time of lung and dLNs were 10 months and 8 months, respectively (4, 25). However, there were no difference in survival time between brain metastasis and bone metastasis, which was revelated again that EHM site was not a suitable prognostic indicator for patients with EHMs.

In this present research, the lung was the most common EHM site, and the brain was the least common site, which implied that it is essential to consider lung metastasis when patients with CC have LM. We also found an interesting result that the primary location of the right tended to have EHMs than of the left, and it accounted for the highest rate (88%) of brain metastasis, which was higher than other metastasis sites. The difference might be induced by the different features between right-sided CC and left-side CC, including molecular, embryological, biological, and anatomical characteristics (26). Research also demonstrated that the sidedness of CC not only has an essential role in the metastatic setting but also is a predictive marker of response to anti-EGFR drugs (26, 27). Previous studies also found that right-sided CC has a more advanced stage at diagnosis (7, 28). In addition, Price et al. speculated that it was induced by the delay in diagnosis for right-sided cancer, which could lead to more metastasis, resulting in worse survival in patients with right-sided cancer (29). Moreover, it is notable that patients with lung metastasis had different frequencies for grade compared with patients with others metastasis. Grade III+IV accounted for only 23% of patients with lung metastasis. However, the rates of grade III+IV were 47, 43, and 42% in patients with metastases of bone, brain, and dLNs, respectively. This result indicated that the occurrence of lung metastasis for patients with LM might not be associated with the level of pathological grade. Li et al. also found that CC patients with lung metastasis were mainly presented as a well-differentiated grade rather than poorly differentiated grade, which is in line with our result (30). However, the detailed mechanism is not revealed, and a string of research is still being urged for this issue.

Based on our results, we concluded that the number of sites of EHMs was a significant predictive factor for CC patients with LM, while the sites of EHMs showed limited impact on survival. Therefore, we constructed a nomogram to predict the survival probability of patients with EHMs. Some clinical features, especially the EHMs number, were involved in this nomogram. Additionally, our nomogram demonstrated strong predictive capacity for the clinical outcome of patients with EHMs in both the training group and the validation cohort. To our best research, it was the first study that aimed to construct a nomogram based on EHMs number. In addition, compared with other research which constructed a prognostic model for CC patients with stage IV, our model also had an obvious advantage. Deng et al. constructed a nomogram model to predict survival time in patients with CRC hepato-pulmonary metastasis. The AUC values of 1 and 3 years were 0.802, 0.759 in the training cohort; 0.814, 0.769 in the validation cohort (31). Han et al. also constructed a model, and the AUC for 1 year was 0.705, for 2 years was 0.675, and for 3 year was 0.648 (32). All these were not excellent as our model, based on that AUC were 0.863, 0.833, 0.815, 0.8, and 0.784 in predicting 6-, 12-, 18-, 24-, and 36- month OS in the training cohort, and 0.862, 0.833, 0.817, 0.807, and 0.792 in a validation cohort.

## Conclusion

In conclusion, the number of sites of EHMs was a significant prognostic factor of CC patients with LM. Patients with zero or one involved extrahepatic organ exhibited better survival compared with patients with two or more EHM sites. Patients with a more significant number of ECMs presented a higher cumulative incidence rate of CSS. Moreover, patients with various EHM sites had different impacts on survival and presented variant distribution features of primary location site, grade, and histology. Finally, a nomogram based on the number of sites of EHMs was constructed that can accurately predict the OS of patients with EHMs.

## Data availability statement

The original contributions presented in the study are included in the article/supplementary material. Further inquiries can be directed to the corresponding author.

## Author contributions

JR supervised the study; SB conceived the study. JR, SB, LC, GZ, YLY, WX, WaL, WeL, FH, NL, MC, and YPY analyzed data; SB wrote the manuscript; JR and SB made manuscript revisions. All authors have read and approved the final version of this submission.
